# Pulmonary Metastases After Surgical Resection of Pancreatic Ductal Adenocarcinoma: Impact on Long-Term Survival and Perspectives on Targeted Treatments

**DOI:** 10.1245/s10434-026-19586-1

**Published:** 2026-04-27

**Authors:** Melroy D’Souza, Malin Andrée, Allan Feili, Omid Sadr-Azodi, Marcus Holmberg

**Affiliations:** 1 Department of Clinical Science, Intervention and Technology, Karolinska Institutet, Stockholm, Sweden; 2 Department of Upper Gastrointestinal Diseases, Karolinska University Hospital, Stockholm, Sweden; 3 Department of Surgery and Oncology, Capio St Gorans Hospital, Stockholm, Sweden

## Abstract

**Background:**

Recurrence impacts outcome after resection for pancreatic ductal adenocarcinoma (PDAC). However, isolated pulmonary metastases exhibit favorable overall survival (OS). Outcomes for patients with pulmonary metastases and concurrent recurrence at other sites are not well studied. This study aimed to assess OS for patients with differing pulmonary recurrence patterns after surgery for PDAC.

**Methods:**

The study included adult patients with PDAC resected between 2009 and 2018 at Karolinska University Hospital. The following three recurrence patterns occurring within 3 years after surgery were compared: metastases involving the liver (liver), pulmonary metastases not involving the liver (lung), and metastases involving neither lung nor the liver (other). Survival analyses with flexible parametric regressions and the Kaplan-Meier method were undertaken.

**Results:**

Of 343 patients included in the study, 293 (85%) experienced recurrence within 3 years. The recurrences included 145 (49%) to the liver, 61 (21%) to the lung*,* and 87 (30%) to other sites. The median OS values for the mentioned groups were 13 months (95% confidence interval [CI], 12–16 months), 30 months (95% CI, 27–35 months), and 20 months (95% CI, 19–26 months) respectively (*p* ≤ 0.001 for all). Flexible parametric survival regressions showed distinct recurrence-specific mortality risks. Compared with no recurrence, the mortality conferred by the lung was 7-fold higher, by other was 12-fold higher, and by the liver was 23-fold higher (*p* ≤ 0.001 for all), demonstrating a clear prognostic hierarchy.

**Conclusions:**

Pulmonary metastases, a frequent recurrence pattern after PDAC surgery, exhibit superior OS compared with other sites, even if combined with peritoneal or other distant spread. This finding supports further consideration of targeted treatment strategies for this subgroup.

Pancreatic ductal adenocarcinoma (PDAC) ranks as the fourth most common cause of cancer-related death in the United States.^1^ Although it is relatively uncommon, and despite advancements in multidisciplinary treatment and postoperative adjuvant chemotherapy,^1,2^ it is projected to become the second leading cause of cancer-related deaths worldwide within the next decade.^3^

Half of the patients with initially resectable disease experience metastases within 1 year, and most experience disease progression within 2 years^4^ resulting in high mortality.^5,6^ The liver is the most common site of early recurrence, occurring in 75 to 90% of patients with disseminated PDAC, followed by locoregional, peritoneal, and pulmonary recurrences.^7–9^ Patients with liver and/or peritoneal recurrence have worse overall survival (OS) than those with pulmonary metastasis, which typically progresses more slowly.^9^ Pulmonary metastasis occurs in about 20 to 25% of cases, and when confined to the lungs only, seen in 5 to 15% of cases,^10,11^ is associated with improved OS.^12^

Various hypotheses attempt to elucidate the factors contributing to why PDAC patients with pulmonary metastases have better OS.^13^ These include a more indolent tumor cell population^14^ and a more active tumor immune response.^15^ Such characteristics are associated with lower tumor stage, reduced vascular invasion, and more differentiated tumor architecture,^12^ all of which influence the typically slower progression of pulmonary metastases and their later onset of recurrence.^16,17^ Notably, the lungs are the most common site for recurrence more than 3 years after pancreatectomy.^8^

Despite advances in understanding the molecular profile, clinical progression, and recurrence patterns of PDAC during the past decade, it remains one of the deadliest cancers.^18^ Although the disease exhibits considerable tumor heterogeneity,^19,20^ current adjuvant therapy still is rather homogeneous and should be updated.^14^ Surgical resection of local recurrence or isolated pulmonary metastasis is occasionally performed,^13,21^ but is not standard practice. Similarly, although selective treatment for peritoneal carcinomatosis has remained controversial, there is growing interest in revisiting this approach.^22,23^

Although pulmonary metastases account for one fifth of PDAC recurrences,^10^ limited research has addressed outcomes for patients who have pulmonary metastasis with other concurrent recurrence sites. This study aimed to investigate recurrence patterns of pulmonary metastases after surgery for resectable PDAC and to compare outcomes for each pattern. We hypothesized that patients with isolated pulmonary metastases have a better outcome than those with additional sites.

## Materials and Methods

This retrospective single-center observational study included all adult patients (age ≥18 years) residing in the Stockholm area (population of 2.4 million) who had PDAC resection between 1 January 2009 and 31 December 2018 at the Karolinska University Hospital, the only tertiary referral center for pancreatic surgery in Stockholm, Sweden. The exclusion criteria ruled out patients not residing in the Stockholm region (because data regarding recurrence were commonly missing), patients who had received neoadjuvant chemotherapy before surgery (i.e., for tumors with arterial and/or advanced venous involvement), and patients deceased within 3 years after resection without apparent disseminated disease. The last follow-up evaluation was 31 December 2023, warranting a follow-up evaluation after a minimum of 5 years for non-deceased patients. Data were extracted from electronic health records. The study was approved by the local Ethical Committee of Stockholm (registration no. DNr 2019-00645).

### Staging, Surgical Procedure, and Pathologic Examination

All the patients were discussed in a multidisciplinary team conference after having undergone multi-phase computed tomography (CT) with pancreatic and pulmonary protocols. The primary tumor was resected using standard procedures, including partial pancreatoduodenectomies and left and total pancreatectomies. Standard lymphadenectomy was performed, and for pancreatoduodenectomy and total pancreatectomy, additional para-aortal lymph node resection was performed on a routine basis. The surgical specimen was examined untouched, with strict adherence to the Leeds Pathology Protocol,^24^ which is known to increase reported positive margins up to 85% without affecting long-term survival.^25^ The staging of PDAC was based on the eighth edition of the American Joint Committee on Cancer’s (AJCC) tumor-node-metastases (TNM) classification.^26^

### Adjuvant Chemotherapy, Follow-up Evaluation, Management of Recurrences, and Definitions

All patients eligible for adjuvant chemotherapy were considered. Treatment began within 3 months after surgery and was considered completed if received during fully 6 months, regardless whether dose reduction occurred. Treatment regimens included gemcitabine monotherapy in combination with capecitabine or nab-paclitaxel or other regimens such as FOLFIRINOX. Adjuvant chemotherapy was considered incomplete if terminated prematurely due to adverse events or if recurrence was diagnosed within the 6 month treatment window, even if chemotherapy subsequently continued as palliative treatment. No patients received radiotherapy.

The institution did not have a structured radiologic follow-up evaluation during the study cohort, but CT scan of the abdominal and thoracic cavities generally was performed annually up to 5 years after surgery, or earlier when motivated. Recurrent disease was determined based on cross-sectional imaging and defined as an occurrence of at least one suspected malignant locoregional, peritoneal, hepatic, pulmonary, skeletal, and/or cerebral lesion after surgery. Histologic confirmation was not routinely performed. Recurrence-free survival (RFS) was defined as the time from the initial pancreatic surgery to the date of recurrent disease.

Recurrences generally were managed with palliative chemotherapy or best supportive care depending on patient and tumor characteristics. Palliative chemotherapy generally was not initiated for asymptomatic pulmonary metastasis with low tumor burden. Resection for local recurrence was performed on rare occasions (*n* = 1), but only if isolated and stationary after some months of chemotherapy.

Tumors that had no apparent recurrence within 3 years, which reportedly accounted for only a minority of resections and that had a lesser negative impact on OS^27^ were in these analyses considered non-recurrent disease. Overall survival was defined as the time from the initial pancreatic surgery to death or last follow-up evaluation, whichever came first.

### Collected Data and Covariates

Descriptive statistics of pre-, peri- and postoperative clinicopathologic characteristics were used. Covariates included gender, age, comorbidities, radiologic tumor size and localization, surgical procedure, TNM classification, pathologic predictors including vascular invasion and surgical margin, vascular resection, tumor type, adjuvant chemotherapy, and recurrence patterns (site, single/multiple sites, time to recurrence). Hereditary gynecologic cancers were defined as those experienced by first-degree relatives with a history of endometrial, ovarian carcinoma, or both.

**Fig. 1:**
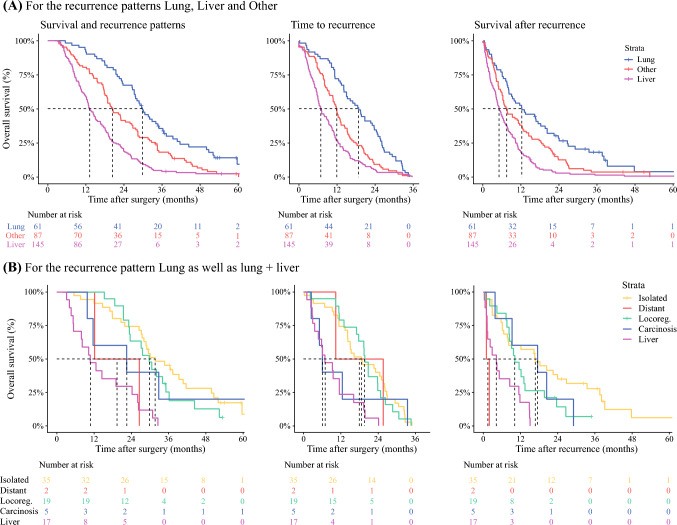
Kaplan-Meier survival analyses. **A** For the lung, liver, and other recurrence patterns. **B** For the recurrence pattern, lung as well as lung and liver. Kaplan-Meier survival analyses. Overall survival, recurrence-free survival, and post-recurrence survival for patients who had recurrence within 3 years, including (**A**) liver, lung, other recurrence, (**B**) with lung recurrence (isolated, distant, locoregional), carcinosis, and liver (for comparison)

After exploratory visual analyses using the Kaplan-Meier survival curves (Fig. 1), all pulmonary metastasis not involving the liver (lung) demonstrated similar OS and were grouped together in further analyses. This group was compared with all metastases involving the liver (liver) as well as metastases involving neither the lung nor the liver (other). Hence, three mutually exclusive groups were retrieved and further compared: lung, liver, and other.

### Statistical Analysis

Numeric data were reported using the median and interquartile range (IQR). Continuous data were presented as absolute count (*n*) and percentage (%) and categorical data as median and interquartile range (IQR). Groups were compared by chi-square test, Mann–Whitney *U* test, Kruskal-Wallis test, and Fisher’s exact test.

### Multivariable Regressions

To identify potential predictors of recurrence patterns, multivariable logistic regression analyses were performed. Potential predictors of recurrence patterns were screened using univariable logistic regression, with a *p* value lower than 0.05 used as the threshold for inclusion, and the candidate variables were entered into full multivariable models. Final parsimonious models were selected using the Akaike Information Criterion (AIC) through a stepwise algorithm allowing both backward and forward steps.

To identify potential predictors of death, multivariable Cox regression was performed, but the proportional hazards assumption was, according to Schoenfeld’s residuals and log-minus-log plots, violated for all recurrences and age. To address these violations, flexible parametric survival regression models with restricted cubic splines (2 internal knots), accommodating non-proportional baseline hazards while maintaining proportional covariate effects,^28^ were fitted. Unadjusted (recurrence pattern only) and fully adjusted models were constructed. Candidate variables included demographic, clinical, tumor-related, and treatment-related factors selected by forward AIC-based selection (ΔAIC ≥2). Characteristics identified as predictors of recurrence site in logistic regressions were excluded because they were hypothesized to mediate survival effects through recurrence pattern. Hazard ratio stability between models was evaluated, with less than 10% variation considered as evidence of minimal confounding.

The effect of covariates on the outcome was calculated and presented as odds ratio (OR) for the logistic and hazard ratio (HR) for the flexible parametric regressions, respectively, including 95% confidence interval (CI). All statistical analyses were performed using R version 4.5.0 GUI, R Foundation for Statistical Computing, Vienna, Austria, 2025.

### Kaplan-Meier Analyses

Median OS, RFS, and post-recurrence survival (PRS) were estimated using the Kaplan-Meier method. Overall survival times were calculated from the date of surgery to the date of death or last follow-up evaluation (whichever came first), RFS times from the date of surgery to the date of first recurrence, and PRS times from recurrence to death or last follow-up evaluation. Pair-wise comparisons between recurrence pattern subgroups (lung, liver, and other) were performed using the log-rank test. Median OS times and survival rates at 1, 3, and 5 years were reported with corresponding 95% CI. Statistical significance was defined as a *p* value lower than 0.05. Kaplan–Meier survival curves were generated to visualize differences in OS, RFS, and PRS across recurrence patterns (Fig. 1a), with additional stratification based on pulmonary recurrence characteristics (Fig. 1b).

## Results

### Baseline Characteristics

Of the 343 patients included in the study, 293 (85%) experienced recurrence within 3 years. Of these 293 patients, 145 (49%) had recurrence to the liver, 61 (21%) had recurrence to the lung, and 87 (30%) had recurrence to other sites (Table 1). Half of the patients were women. The median age was 70 years. One third of the women had an American Society of Anesthesiology (ASA) score higher than 2. About a one fourth were comorbid with diabetes mellitus, but there were no differences between the groups. Three fourths of the patients had CA19-9 levels of ≥37 U/ml.

**Table 1:** Baseline and radiologic characteristics

Recurrence							
Variables	Overall (*n* = 293) *n* (%)	Lung (*n* = 61) *n* (%)^a^	Other (*n* = 87) *n* (%)^a^	Liver (*n* = 145) *n* (%)^a^	*p* Value^c^	*p* Value^d^	*p* Value^*5*e^
Sex					0.500	0.284	0.405
Female	129 (44)	31 (51)	37 (43)	61 (42)			
Male	164 (56)	30 (49)	50 (57)	84 (58)			
Age: years (range)	70 (64–75)	68 (63–75)	69 (62–74)	71 (65–75)	0.162	0.114	0.832
ASA					0.630	>0.999	0.487
1–2	184 (63)	37 (61)	58 (67)	89 (61)			
3–4	109 (37)	24 (39)	29 (33)	56 (39)			
BMI					0.192	0.273	0.738
<25	177 (60)	35 (57)	47 (54)	95 (66)			
≥25	116 (40)	26 (43)	40 (46)	50 (34)			
Heart disease	144 (49)	23 (38)	49 (56)	72 (50)	0.071	0.128	**0.030**
Smoking	108 (37)	24 (40)	34 (39)	50 (34)	0.675	0.523	>0.999
Diabetes mellitus					**0.045**	0.224	0.146
New onset	9 (3.1)	4 (6.6)	1 (1.1)	4 (2.8)			
Worsening	20 (6.8)	3 (4.9)	2 (2.3)	15 (10)			
History of cancer	66 (23)	9 (15)	21 (25)	36 (25)	0.262	0.140	0.154
Gastrointestinal	13 (4.4)	1 (1.6)	4 (4.6)	8 (5.5)	0.483	0.286	0.401
Urinary	26 (8.8)	4 (6.6)	7 (8.0)	15 (10)	0.642	0.598	>0.999
Heredity of gyn cancer	19 (6.8)	7 (12)	8 (9.5)	4 (2.9)	**0.028**	**0.020**	0.783
CA19-9 (U/mL)					0.711	0.575	0.542
≤37	61 (22)	15 (25)	17 (20)	29 (21)			
>37	221 (78)	44 (75)	67 (80)	110 (79)			
Location					0.800	0.776	0.495
Head	229 (78)	46 (75)	68 (78)	115 (79)			
Body	30 (10)	6 (9.8)	11 (13)	13 (9.0)			
Tail	32 (11)	9 (15)	7 (8.0)	16 (11)			
Extensive	2 (0.7)	0 (0)	1 (1.1)	1 (0.7)			
Procedure					0.900	0.594	0.851
Pancreatoduodenectomy	201 (69)	39 (64)	59 (68)	103 (71)			
Left pancreatectomy	54 (18)	13 (21)	16 (18)	25 (17)			
Total pancreatectomy	38 (13)	9 (15)	12 (14)	17 (12)			
Tumor size: mm (range)	26 (20–33)	25 (20–30)	29 (20–35)	26 (20–33)	0.695	0.382	0.664
Venous contact	105 (36)	24 (39)	35 (40)	47 (33)	0.463	0.422	>0.999

ASA - American society of anesthesiology; BMI - Body mass index; gyn - Gynecologic

^a^Median (25–75%)

^b^Fisher’s exact test, Kruskal-Wallis rank-sum test, Pearson’s chi-square test

^c^All three groups compared: Fisher’s exact test, Wilcoxon rank-sum test, Pearson’s chi-square test

^d^Lung vs. liver: Fisher’s exact test, Wilcoxon rank-sum test, Pearson's chi-square test

^e^Lung vs. other: Fisher’s exact test, Wilcoxon rank-sum test, Pearson’s chi-square test

The tumor was solid in most cases, situated in the head in four fifths of the cases, measured 36 mm, and usually did not display any vein involvement. Venous resection was deemed necessary in one third of the cases. Most tumors were primarily stage T2 and had poorly differentiated or undifferentiated histology. More than half of the patients had N2-stage lymph node involvement as well as more than two thirds of those with pulmonary metastases.

Although single-site recurrences were common overall, 43% and 44% of those with lung and liver metastases, respectively, experienced multiple-site recurrences compared with 22% of those with other recurrences. Among the three patient cohorts with recurrence presented in Table 2, fewer patients with pulmonary metastases showed metastatic disease progression at 1 and 2 years.

**Table 2:** Pathologic characteristics and recurrence patterns

Recurrence							
Variables	Overall (*n* = 293) *n* (%)	Lung (*n* = 61) *n* (%)^a^	Other (*n* = 87) *n* (%)^a^	Liver (*n* = 145) *n* (%)^a^	*p* Value^b^	*p* Value^c^	*p* Value^d^
Tumor size: mm (range)	38 (30–46)	40 (30–48)	36 (30–47)	39 (30–45)	0.899	0.649	0.727
Differentiation					**<0.001**	**<0.001**	0.449
Well or moderate	98 (33)	34 (56)	39 (44)	26 (18)			
Poor or undifferentiated	194 (67)	27 (44)	48 (56)	118 (82)			
Subtype					**0.022**	**0.020**	0.872
Pancreatobiliary	151 (83)	33 (92)	46 (90)	72 (77)			
Intestinal	7 (3.9)	2 (5.6)	2 (3.8)	3 (3.2)			
Adenosquamous	23 (13)	1 (2.8)	3 (5.8)	19 (20)			
T stage					0.833	0.517	0.741
1	5 (1.7)	1 (1.6)	2 (2.3)	2 (1.4)			
2	171 (58)	32 (52)	52 (60)	87 (60)			
3	117 (40)	28 (46)	33 (38)	56 (39)			
N stage					0.443	0.212	0.387
0–1	111 (38)	19 (31)	33 (38)	59 (41)			
2	182 (62)	42 (69)	54 (62)	86 (59)			
M1-stage	56 (19)	11 (18)	20 (23)	25 (17)	0.571	>0.999	0.543
Microscopic invasion							
Lymphovascular	261 (89)	56 (92)	77 (89)	127 (88)	0.741	0.622	0.591
Vascular	232 (79)	49 (80)	65 (74)	118 (81)	0.377	0.848	0.434
Perineural	275 (95)	55 (90)	81 (94)	139 (98)	**0.030**	**0.023**	0.553
Surgical margin	244 (83)	50 (82)	74 (85)	119 (82)	0.587	0.457	0.628
Adjuvant Chemotherapy	173 (61)	47 (77)	50 (60)	76 (54)	**0.010**	**0.003**	**0.047**
Recurrence							
Single site	184 (63)	35 (57)	69 (78)	81 (56)	**0.002**	0.823	**<0.001**
Lung	35 (12)	35 (57)	0 (0)	0 (0)			
Locoregional	42 (14)	0 (0)	42 (48)	0 (0)			
Carcinosis	21 (7)	0 (0)	21 (24)	0 (0)			
Misc^e^	5 (1.7)	0 (0)	5 (5.7)	0 (0)			
Liver	81 (28)	0 (0)	0 (0)	81 (56)			
Multi-site	109 (37)	26 (43)	19 (22)	64 (44)	**0.002**	0.879	**0.007**
Lung	43 (15)	26 (43)	0 (0)	17 (12)			
Locoregional	83 (28)	21 (34)	19 (22)	43 (30)			
Carcinosis	40 (14)	5 (8)	14 (16)	21 (14)			
Misc^e^	8 (3)	4 (7)	0 (0)	4 (3)			
Liver	64 (22)	0 (0)	0 (0)	64 (44)			

^a^Median (25–75%)

^b^All three groups compared: Fisher’s exact test; Wilcoxon rank sum test; Pearson’s chi-square test

^c^Lung vs. liver: Fisher’s exact test, Wilcoxon rank-sum test, Pearson’s chi-square test

^d^Lung vs. other: Fisher’s exact test, Wilcoxon rank-sum test, Pearson’s chi-square test

^e^Skeleton, brain

### Overall Survival

The median OS was 22.8 months (95% CI, 19.9–25.9 months) for the entire study population, 18.8 months (95% CI, 17.5–20.5 months) for the surviving patients who experienced recurrence within 3 years, and 84.6 months (95% CI, 62.1–119.0 months) for the patients who had no recurrence within 3 years. The patients with lung metastases had significantly longer median OS (29.8 months; 95% CI, 27.2–35.0 months) than those with liver recurrences (13.3 months; 95% CI, 12.2–15.6 months; *p* < 0.001) and those with other recurrences (20.4 months; 95% CI, 18.5–26.4 months; *p* < 0.001).

### Recurrence-Free Survival

For recurrent disease, RFS was 10.9 months (95% CI, 9.44–11.7 months). The patients with lung metastases had significantly longer RFS (18.8 months; 95% CI, 15.0–23.8 months) than those with liver metastases (6.90 months; 95% CI, 6.08–9.21 months; *p* < 0.001) and those with other recurrences (11.9 months; 95% CI, 10.7–13.3 months; *p* < 0.001).

### Post-Recurrence Survival

For recurrent disease, PRS was 7.04 months (95% CI, 6.12–8.15 months). The patients with lung metastases had significantly longer PRS (12.2 months; 95% CI, 8.35–19.7 months) than those with liver metastases (5.13 months; 95% CI, 4.27–6.74 months; *p* < 0.001) and those with other recurrences (7.59 months; 95% CI, 6.35–11.6 months; *p* < 0.001).

### Kaplan-Meier Survival Curves

Kaplan-Meier survival curves for OS as well as for RFS and PRS according to different recurrence patterns are presented in Fig. 1a. Corresponding survival curves stratified based on pulmonary recurrence patterns are presented in Fig. 1b.

### Predictive Covariates for Pulmonary Metastases and Death

In assessing the clinical factors that contribute to the development of metastases and recurrence in PDAC patients, our multivariable logistic regression (Table 3A) indicated that undifferentiated or poorly differentiated tumors were predictive for liver metastases, that well-differentiated or moderately differentiated tumors were predictive for both lung and other recurrences, that new-onset diabetes mellitus or worsened existing diabetes mellitus was predictive for liver metastasis but protective against other recurrences, that completed adjuvant chemotherapy was predictive for lung recurrences, and that heredity for gynecologic cancer was protective for liver recurrence (Table 3A).

**Table 3:** Multivariable regressions for recurrence patterns within 3 years and survival

A Logistic regressions: predictors of recurrence site^a^									
	Lung			Other			Liver		
Covariates	OR	95% CI	*p* Value	OR	95% CI	*p* Value	OR	95% CI	*p* Value
*Heredity*, *gyn*. *Cancer*									
No	—	—		—	—		Ref		
Yes	—	—		—	—		0.18	0.05–0.68	**0.013**
*Diabetes mellitus*									
No DM, or stable	—	—		Ref			Ref		
New onset, or worsened	—	—		0.23	0.07–0.79	**0.022**	2.66	1.04–6.79	**0.040**
*Tumor differentiation*									
Well or moderate	Ref			Ref			Ref		
Poor or undifferentiated	0.33	0.18–0.61	**<0.001**	0.45	0.26–0.78	**0.005**	5.40	2.90–10.1	**<0.001**
*Adjuvant chemotherapy*									
Not initiated	Ref			—	—	—		—	
Completed	2.20	1.12–4.33	**0.023**	—	—		—	—	

**B** Parametric flexible regressions for death, presenting significant predictors^b^						
	Unadjusted			Adjusted		
Covariates	HR	(95% CI)	*p* Value	HR	(95% CI)	*p* Value
Recurrence			**<0.001**			**<0.001**
None	Ref.			Ref.		
Lung	7.81	4.69–13.0		7.53	4.52–12.5	
Other	12.5	7.58–20.5		11.9	7.23–19.7	
Liver	24.0	14.7–39.2		23.0	14.1–37.4	
Age (per 10 year increase)	—	—		1.23	1.07–1.41	**0.003**
Adjuvant chemotherapy						**<0.001**
Not initiated or completed	—	—		Ref.		
Completed	—	—		0.58	0.45–0.75	

OR Odds ratio, CI Confidence interval,—, not included in final model, Ref Reference category, DM Diabetes mellitus

^a^Model fit statistics: among 293 patients who had recurrence within 3 years, 258 with complete data were included in analyses (57 lung, 77 other, 124 liver). Models were built using forward selection based on Akaike Information Criterion (AIC). Model fit: lung (AIC, 259; pseudo *R*^2^, 0.07); other (AIC, 305; pseudo *R*^2^, 0.04); liver (AIC, 321; pseudo *R*^2^, 0.12). Variance inflation factors (VIF) < 2 for all variables, indicating no multicollinearity

HR Hazard ratio, CI Confidence interval;—, not in unadjusted model

^b^Model statistics: of a total of 352 patients (293 with and 59 without recurrence), the flexible parametric survival analysis included 342 patients (295 deaths, 47 censored). After exclusion of 10 patients due to missing data, the analysis had 342 patients (295 events, 47 censored). Flexible parametric model had 2 internal knots. Recurrence site HRs showed <5% variation between models. Unadjusted AIC, 2420; adjusted AIC, 2358 (ΔAIC,–62)

Flexible parametric regression analysis showed distinct risk profiles for death (Table 3B). In the fully adjusted model, hazard ratios compared no recurrence with lung (HR, 7.53; 95% CI, 4.52–12.5)*,* liver (HR, 23.0; 95% CI, 14.1–37.4), and other (HR, 11.9; 95% CI, 7.23–19.7) recurrence (*p* < 0.001), whereas the hazard ratios were 1.23 (95% CI, 1.07–1.41; *p* = 0.003) for age (with 10 year increments) and 0.58 (95 CI, 0.45–0.75; *p* < 0.001) for completed adjuvant chemotherapy. Variation between unadjusted and full adjusted models was less than 5%. The AIC improved from 2454 to 2426.

## Discussion

This observational study from a high-volume tertiary pancreatic center investigated OS for patients who experienced pulmonary metastases after surgery for upfront resectable PDAC and compared the outcomes with those for patients with other recurrence patterns. We found that lung recurrence exhibited a significantly longer OS than either liver metastases and other recurrences, suggesting that patients with pulmonary recurrences, even with synchronous spread outside the liver, may have a better prognosis and may still benefit from individualized treatment strategies.

Recurrence after PDAC surgery markedly curtails survival, particularly with liver and/or peritoneal involvement, whereas pulmonary metastases correlate with improved outcome, especially if isolated.^12,29,30^ These observations align with our results, in which median OS varied substantially by pattern. Pulmonary-directed therapies, such as resection or ablation, have doubled median OS compared with current treatment (68 vs. 34 months and 51 vs. 23 months),^13,31^ with a meta-analysis of 467 patients confirming a pooled HR of 0.64 favoring metastasectomy over chemotherapy.^32^Positive factors include RFS shorter than 18 months, unilateral/few metastasis, and low/reduced CA19-9 levels.^10^ Thus, local treatment for distant pulmonary metastases improves OS, suggesting an indolent and therapy-susceptible cell population in pulmonary metastases.

The favorable outcomes for lung metastases extend beyond isolated pulmonary disease to certain combined recurrence patterns. Local treatment also has been used for select patients with locoregional recurrence after PDAC surgery in the form of re-resection after chemotherapy.^33^ In a systematic review and meta-analysis of 431 patients, the median OS increased 15.2 months after re-resection, and the authors concluded that selection of patients and assessment of time and site of recurrence are mandatory.^34^ Interestingly, patients with pulmonary metastases and simultaneous locoregional recurrence in our study experienced survival comparable with that of isolated pulmonary metastases in the first years.

Moreover, patients with pulmonary recurrence and concurrent peritoneal carcinomatosis also showed OS comparable with that of isolated pulmonary recurrence in our study. This is surprising because peritoneal carcinomatosis typically portends a poor prognosis^35^ and is increasingly diagnosed,^36^ yet our data suggest that it harbors indolent biology when it occurs in combination with pulmonary spread, displaying a relatively promising outcome. To our knowledge, this is the first report of a favorable OS for patients with such synchronous recurrence patterns. This observation, although based on a small subset (*n* = 5), warrants validation in further studies and larger cohorts. If confirmed, patients with this recurrence pattern may represent candidates for more aggressive multimodal strategies potentially including cytoreductive surgery (CRS) with or without addition of intraperitoneal chemotherapy.^22,37–39^

In single-organ recurrences, RFS is a strong predictor for OS, suggesting its potential utility in patient selection for lung-directed therapies.^40–42^ In our cohort, the lung subgroup showed notably longer median RFS (19 vs. 12 months for other and 7 months for liver). The median duration of PRS was significantly longer in the lung recurrence group (11.9 months) than in the liver (5.14 months) and other (7.65 months) recurrence groups. These findings align with prior studies reporting longer PRS in lung-only recurrences (15.4 vs. 9.7 months or shorter; *p* ≤ 0.001),^27^ and even greater benefit after pulmonary metastasectomy (30.8 vs. 18.6 months; *p* < 0.01).^43^

These differences suggest that pulmonary recurrences, even when accompanied by peritoneal or distant spread, may reflect a more indolent tumor biology. This highlights the importance of tailoring treatment strategies for recurrent PDAC based on anticipated survival outcomes, which vary significantly across patient subgroups. Such variation emphasizes the critical need for individualized interventions, accounting for individual tumor behavior as well as patient-specific prognostic factors and that such interventions are precisely timed. Notably, the median OS advantage and visual patterns in Kaplan-Meier curves suggest a potential therapeutic window of 18 to 24 months after resection (Fig. 1), when most lung metastases have been detected but remain confined to favorable sites. Delayed intervention may risk progression beyond this more favorable biologic phase.

Our multivariable analyses showed a clear biologic hierarchy determining survival outcomes. In logistic regression (Table 3A), tumor differentiation strongly predicted metastatic site, with poor differentiation associated with liver metastases and well-differentiated tumors preferentially metastasizing to the lung or other sites. In flexible parametric survival regressions (Table 3B), recurrence site was the dominant predictor of mortality, with liver recurrence conferring a threefold worse survival than lung metastases. These findings suggest that tumor biology influences survival primarily through its determination of metastatic organotropism: well-differentiated tumors preferentially metastasize to the lung where they confer better prognosis, whereas poorly differentiated tumors favor liver metastases, with significantly worse outcomes.

The stability of recurrence-site hazard ratios between the unadjusted and adjusted flexible parametric survival regression models (<5% variation) demonstrates that survival differences are independent of age and treatment received, reflecting fundamental differences in tumor-organ interactions. This aligns with Paget´s seed-and-soil hypothesis positing that metastatic success depends on compatibility between metastatic tumor cells and the microenvironment of the target organ.^44^ This seed-and-soil distinction for lung metastases marks a biologically and clinically meaningful difference, supporting intensive surveillance for early detection and aggressive locale therapies for selected patients with lung recurrence, recognizing that although not curative, such interventions may substantially prolong survival.

Despite the tumoral heterogeneity characterized in PDAC,^19,20^ current oncologic practice tends to treat metastatic disease rather homogeneously. Recurrence typically is managed with systemic chemotherapy, offering a limited number of pharmacologic alternatives, either at diagnosis or upon the onset of symptoms. However, our findings suggest potential for a paradigm shift, leading to new treatment approaches for patients with pulmonary metastasis in highly selected cases and in investigational regimens. This could include pulmonary metastasectomy, re-resection of the pancreatic remnant, CRS, and/or intraperitoneal chemotherapy for peritoneal disease, as well as novel systemic therapeutic options, even for cases in which recurrence is not confined to the lungs.

Emerging systemic options target specific pathways based on genomic alternations^45^ and molecular PDAC subtypes.^14^ In our cohort, family history of gynecologic cancer was independently protective against liver recurrence. These gynecologic cancers share germline risk factors with PDAC, including pathogenic variants in BRCA1/2, PALB2, and Lynch syndrome genes.^6^ Intriguingly, PDAC with lung-only versus liver-only metastasis exhibits distinct genomic alterations and outcomes,^7^ suggesting that germline factors may influence organ-specific colonization. Although our definition relied on family history rather than confirmed germline testing, this association warrants further investigation with comprehensive genetic analysis.

This study had notable strengths that support the validity of our findings. First, the patient cohort was from a high-volume tertiary pancreatic center with standardized preoperative, surgical, and oncologic protocols, reducing treatment heterogeneity. Second, the long-term follow-up period (>5 years) was near-complete with detailed documentation of recurrence patterns and survival outcomes. Third, our multivariable analyses, including both logistic regression for recurrence predictors and flexible parametric survival models, demonstrated stable effect estimates across models, indicating robust findings independent of potential confounders. Finally, this study was among the first studies to systematically characterize outcomes for combined pulmonary recurrence patterns, addressing an important clinical gap.

Despite these strengths, this study had several important limitations that warrant consideration. First, its retrospective and single-center design introduced inherent biases, including selection bias and limited generalizability to broader patient populations. Additionally, the curated clinical data may have been prone to inaccuracies or incomplete documentation. Second, the exclusion of patients who died within 3 years without documented recurrence may have introduced an immortal time bias, potentially overestimating survival in the non-recurrent group. Third, the absence of a standardized radiologic follow-up protocol and routine histologic confirmation may have influenced both the timing and accuracy of recurrence detection. Moreover, variability in chemotherapy administration, particularly in real-world settings, could have affected both recurrence patterns and RFS as well as survival after recurrence. However, given that asymptomatic pulmonary metastases generally did not receive palliative chemotherapy, a positive effect on survival was less likely. Importantly, this study was not designed to assess chemotherapy efficacy, but rather to explore outcome for pulmonary recurrence patterns. Our interpretation of the findings reflects this focus. Finally, the relatively small cohort size should be regarded as exploratory and in need of external validation in future studies, especially regarding certain subgroups with a small number of patients (lung and carcinomatosis, *n* = 7).

In conclusion, our study demonstrated that all recurrence patterns involving the lung, excluding those with concurrent liver involvement, are associated with a relatively favorable prognosis that advocates for aggressive local, regional, and/or systemic intervention. This study is the first to report favorable outcomes for pulmonary metastases combined with peritoneal or other distant spread. Randomized controlled trials studying targeted therapies for these recurrence patterns are urgently needed.
